# Polarization-Independent Circulator Based on Composite Rod of Ferrite and Plasma in Photonic Crystal Structure

**DOI:** 10.3390/nano11020381

**Published:** 2021-02-02

**Authors:** Mi Lin, Lixin Fu, Shakeel Ahmed, Qiong Wang, Yaoxian Zheng, Zixian Liang, Zhengbiao Ouyang

**Affiliations:** 1 THz Technical Research Center of Shenzhen University, Shenzhen Key Laboratory of Micro-Nano Photonic Information Technology, Key Laboratory of Optoelectronic Devices and Systems of Ministry of Education and Guangdong Province, College of Physics and Optoelectronic Engineering, Shenzhen University, Shenzhen 518060, China; linfengas111@szu.edu.cn (M.L.); 2060453057@email.szu.edu.cn (L.F.); shakeelahmed6655@gmail.com (S.A.); qwang@szu.edu.cn (Q.W.); jewel5282@163.com (Y.Z.); 2 Institute of Microscale Optoelectronics, Shenzhen University, Shenzhen 518060, China; zixianliang@szu.edu.cn

**Keywords:** polarization-independent, circulator, ferrite, plasma, photonic crystal

## Abstract

We propose a type of polarization-independent circulator based on a composite rod of ferrite and plasma materials in a two-dimensional photonic crystal (PhC) slab. Only one composite rod was set at the center of the structure to provide circulation for both TE- and TM-polarized waves. Additionally, to improve the performance of the circulator, three additional rods were inserted to improve the coupling condition between the center magneto-optical microcavity and the corresponding waveguides. Finite element method was used to calculate the characteristics of the structure and the Nelder–Mead optimization method was employed to obtain the optimum parameters. The results show that a low insertion loss (~0.22 dB) and high isolation (~14 dB) can be achieved in our structure for waves of both TE and TM polarizations. The idea presented here may be useful for designing compact polarization devices in large-scale integrated photonic circuits.

## 1. Introduction

With the development of photonic crystal (PhC) theory, the properties of PhC have been systematically discussed and studied [1,2]. Plenty of devices based on PhC structure have been designed and realized [3,4,5,6,7,8,9,10,11,12]. As a basic component in modern photonics, PhC circulators have attracted much attention for their micro size and high performance. Circulators are a kind of nonreciprocal device that play an important role in optics and photonics. They can be used for protecting useful signals from harmful reflections, reducing the unwanted wave interferences; they can also be used for extracting feedback signals for detectors or monitors [13,14,15,16,17,18,19,20,21,22].

Up until now, many high-quality circulators have been built based on PhCs, where magneto-optical (MO) materials including ferrite and plasma materials are often introduced into the structure to provide a circulation effect for electromagnetic waves or lights. For example, around 2005, three- and four-port two-dimensional (2D) PhC circulators based on MO materials were first proposed by Fan and Wang [13,14,15]. Coupled mode theory was applied in their designs, and high isolation and transmission circulators were successfully built by coupling an MO cavity and waveguides at optical frequencies. Their designs can be used in TM polarization. Then, improved Y-typed and T-typed structures in three-, four-, and even six-port PhC circulators were further studied by introducing cascaded MO resonance cavities to enlarge the bandwidth of the circulators [16,17,18,19]. These works operated in TE polarization. Furthermore, several kinds of novel low-symmetry circulators were investigated by Jin and Dmitriev [20,21]. A very low splitting factor was used in their design, making their circulators feasible for the microwave or terahertz region. Their designs worked in TE polarization.

Considering the above, it is clear that most of the PhC circulators that have been proposed can only operate in a specified polarization (i.e., TE or TM polarization only), which may set some limitations for their potential applications. Thus, it is necessary to research polarization-independent circulators (PICs).

In our previous study [22], a kind of PIC based on MO materials was investigated. In that work, more than one MO rod was introduced into the central cavity, and the corresponding waveguides and the structure of the central cavity were also modulated so that the overall structure was relatively complicated. In this paper, a novel PIC is proposed, in which only one composite MO rod of ferrite and plasma materials is needed at the central cavity. Moreover, only three additional rods are inserted into the waveguides to improve the performances of both TE and TM polarizations in our optimized model. Compared with [22], the PIC in this paper is simpler and more compact in structure, and it is easier to integrate with other devices. To our best knowledge, this is the first realization of the PIC in PhC structure with only one composite MO rod of ferrite and plasma materials. Finite element method [23,24,25] was used to calculate the characteristics of the structure and the Nelder–Mead optimization method (NOM) [26,27] was employed to obtain the optimized parameters.

## 2. Physical Model

**Figure 1:**
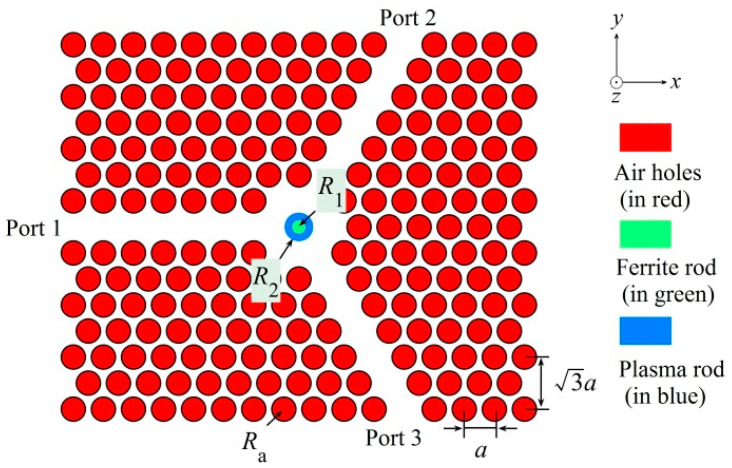
Schematic of the polarization-independent circulator (PIC) structure, where the air holes, ferrite rod, and plasma rod are indicated by red, green, and blue respectively.

The schematic of the PIC is shown in Figure 1. The structure is based on a 2D triangular-lattice PhC, where air holes (in red) were drilled in a silicon slab to form a Y-shaped PhC waveguide. In the central region, a composite MO rod containing ferrite (in green) and plasma (in blue) materials was inserted to construct the center MO microcavity and provide the rotation effect for both TE and TM polarizations. The lattice constant is assumed to be *a*, and the radius of the air hole, the radius of the ferrite rod (in the center of the composite MO rod), and the radius of the composite MO rod are denoted by $R_{a}$, $R_{1}$, and $R_{2}$, respectively. For convenience, a parameter *K* = $R_{1}/R_{2}$ is also defined to be the ratio of the radius of the ferrite rod to that of the composite MO rod.

**Figure 2:**
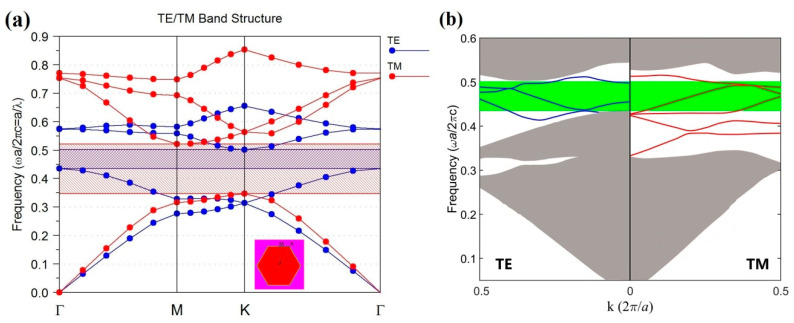
(**a**) Band structure of a perfect 2D triangular-lattice photonic crystal (PhC), where the blue, red, and common regions represent the TE, TM, and absolute photonic bandgap (PBG), respectively. (**b**) The dispersion curves of the guided modes in the input and output waveguides. The blue and red curves indicate the TE- and TM-guided modes, respectively. The green and gray regions indicate the absolute PBG and passband of PhC, respectively.

The refractive indices of silicon slab and air holes were set to be 3.4 and 1. According to previous studies [28,29], complete or absolute photonic bandgap (PBG) can be found in a perfect 2D triangular-lattice PhC by using $R_{a}=0.47a$. The band structure is shown in Figure 2a, where the range of absolute PBG can be found as 0.435–0.501 (*ωa*/2πc). We noted that $R_{a}=0.48a$ corresponded to a wider PBG than $R_{a}=0.47a$. However, after simulation and comparison, we found in this case that the absolute bandgap was near the top edge of the TM-wave PBG, which means that the TM-wave PBG effect in the absolute PBG was weaker than that of the TE-wave PBG. In order to balance the performances of both polarizations, we chose $R_{a}=0.47a$. When the lattice constant a was 10 mm, the range of absolute PBG was from 1.305 to 1.503 $\times 10^{10}$ Hz, which is located in the microwave band. In this range of frequency, the losses of materials used in this paper can be ignored [13,14,15,30,31]. For better understanding of the waveguide mechanism, the dispersion curves of the guided modes in the input and output waveguides are plotted in Figure 2b. From Figure 2b, we can easily obtain the range of the absolute PBG as the region shown in green, fitting quite well with that in Figure 2a. Moreover, the TE- and TM-guided modes can be found in Figure 2b, as shown by the blue and red curves in the absolute bandgap (the green band), respectively. It is seen that the guided modes for both TE and TM waves are all in the absolute PBG region. It should be pointed out that, under the coordinate system as shown in Figure 1, the electric field is parallel to the Z-axis for TE polarization, while the magnetic field is parallel to the Z-axis and the electric field is parallel to the X–Y plane for TM polarization.

In this paper, the ferrite and plasma were chosen to be experimentally feasible materials as follows: Yttrium iron garnet was chosen for the ferrite material. Under an external magnetic field applied in the +Z-direction, the relative permeability of ferrite material can be expressed by a tensor as [16,17,18,19]

(1) $$[\mu_{f}]=\left[\begin{array}{ccc} \mu_{m} & j\mu_{k} & 0\\ -j\mu_{k} & \mu_{m} & 0\\ 0 & 0 & 1 \end{array}\right],$$

where $\mu_{m}=1+\omega_{m}(\omega_{0}+i\alpha \omega )/[{\left(\omega_{0}+i\alpha \omega \right)}^{2}-\omega^{2}]$ and $\mu_{k}=\omega_{m}\omega /[{\left(\omega_{0}+i\alpha \omega \right)}^{2}-\omega^{2}]$ with $\omega_{0}=\mu_{0}\gamma H_{0}$, $\omega_{m}=\mu_{0}\gamma M_{s}$. Here, $\gamma =1.759\;\times \;10^{11}$ C/kg is the gyromagnetic ratio, $\alpha \;=\;3\;\times \;10^{-5}$ is the loss coefficient, and $M_{s}=2.39\;\times \;10^{5}$ A/m is the saturation magnetization. The relative permittivity of ferrite material is given by $\varepsilon_{f}\;=\;12.9$, and the applied magnetic field was set to be $H_{0}\;=\;3.5\;\times \;10^{5}$ A/m. As for the plasma material, the relative permeability is $\mu_{p}\;=\;1$, and under the external magnetic field applied in the +Z-direction, the relative permittivity can be expressed as [30,31]

(2) $$[\varepsilon_{p}]=\left[\begin{array}{ccc} \varepsilon_{m} & j\varepsilon_{k} & 0\\ -j\varepsilon_{k} & \varepsilon_{m} & 0\\ 0 & 0 & \varepsilon_{p} \end{array}\right],$$

where $\varepsilon_{m}=1-\omega_{p}{}^{2}(\omega -jv)/\omega ({\left(\omega -jv\right)}^{2}-\omega_{c}{}^{2})$, $\varepsilon_{k}=-\omega_{c}\omega_{p}{}^{2}/\omega ({\left(\omega -jv\right)}^{2}-\omega_{c}{}^{2})$, and $\varepsilon_{p}=1-\omega_{p}{}^{2}/\omega (\omega -jv)$. Here $\omega_{p}={\left(e^{2}n_{e}/\varepsilon_{0}m\right)}^{1/2}$ is the plasma frequency, where *e*, *m*, and *n*_e_ are the electron charge, electron mass, and plasma density, respectively; $\omega_{c}=\left(eB/m\right)$ is the cyclotron frequency of electron, and *v* is the plasma frequency. When the external magnetic field is $H_{0}\;=\;3.5\times 10^{5}$ A/m, we have *n*_e_ = 10^13^ cm^−3^ and *v* = 1 × 10^−5^*ω*_p_.

In our previous work [22], we proved that the ferrite material can provide a rotation effect for TE polarization, while the plasma material can provide a rotation effect for TM polarization. The mechanism can be understood as follows: For TE polarization, the electric field has only an *E*_z_ component and the magnetic field has both *H*_x_ and *H*_y_ components. When the TE wave transmits through the magnetized ferrite material, the two components of magnetic field *H*_x_ and *H*_y_ will be different in phase due to the imaginary part $\mu_{12}=j\mu_{k}$ and $\mu_{21}=-j\mu_{k}$ of $\left[\mu_{f}\right]$ in the ferrite material (this result can be obtained by solving the wave equation of **H**, and its detailed deduction can be found in [22]), leading to the direction of magnetic field **H** changing along the elliptical path. Therefore, the propagation direction of the wave will change or rotate by noting that the direction of the electric field is fixed along the Z-direction. However, for a TE wave transmitting through the plasma material, there will be no phase difference between *H*_x_ and *H*_y_ because the relative permeability of plasma $\mu_{p}$ is a constant, and so the propagation direction of the wave will remain unchanged. As a result, when the TE wave meets the composite rod containing ferrite and plasma material in our structure, the ferrite part plays the “rotation effect” role and the plasma part plays the “normal effect” role. A similar analysis can be performed for TM polarization, where the roles of ferrite and plasma material are exchanged. When the TM wave meets the composite rod, the ferrite part plays the “normal effect” role and the plasma part plays the “rotation effect” role.

The principle can also be understood by the effective medium theory [32]. According to the effective medium theory, the effective relative permittivity $\left[\varepsilon_{eff}\right]$ and effective relative permeability $\left[\mu_{eff}\right]$ of the composite rod in our structure can be written as

(3) $$[\varepsilon_{eff}]=\left[\begin{array}{ccc} f_{1}(\varepsilon_{f},\varepsilon_{m},R_{1},R_{2}) & f_{2}(\varepsilon_{f},\varepsilon_{m},R_{1},R_{2})+j\cdot f_{3}(\varepsilon_{k},R_{1},R_{2}) & 0\\ f_{2}(\varepsilon_{f},\varepsilon_{m},R_{1},R_{2})-j\cdot f_{3}(\varepsilon_{k},R_{1},R_{2}) & f_{1}(\varepsilon_{f},\varepsilon_{m},R_{1},R_{2}) & 0\\ 0 & 0 & f_{4}(\varepsilon_{f},\varepsilon_{p},R_{1},R_{2}) \end{array}\right],$$

(4) $$[\mu_{eff}]=\left[\begin{array}{ccc} g_{1}(\mu_{f},\mu_{m},R_{1},R_{2}) & g_{2}(\mu_{f},\mu_{m},R_{1},R_{2})+j\cdot g_{3}(\mu_{k},R_{1},R_{2}) & 0\\ g_{2}(\mu_{f},\mu_{m},R_{1},R_{2})-j\cdot g_{3}(\mu_{k},R_{1},R_{2}) & g_{1}(\mu_{f},\mu_{m},R_{1},R_{2}) & 0\\ 0 & 0 & g_{4}(\mu_{p},R_{1},R_{2}) \end{array}\right],$$

where $f_{i}$ and $g_{i}$ (*i* = 1–4) are functions related to the quantities indicated in the parentheses. According to the expressions of effective relative permittivity and effective relative permeability, we can see that the composite rod containing ferrite and plasma materials can provide the rotation effect for both TE and TM polarizations.

## 3. Numerical Results and Discussion

**Figure 3:**
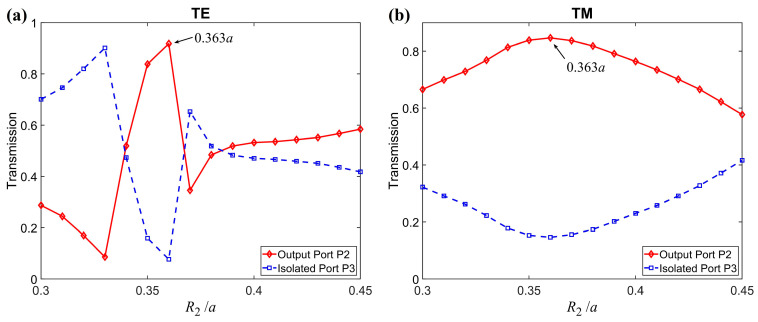
Transmissions of the output port P2 and isolated port P3 versus the radius $R_{2}$ of the composite rod for (**a**) TE and (**b**) TM polarization.

Firstly, considering the important role of the central MO composite rod in the structure, we investigated the influence of the composite-rod radius $R_{2}$ on the performance of the PIC, as shown in Figure 3. Due to the structure symmetry, the properties of the circulator can be studied by choosing an arbitrary port as the input port. Here, we selected P1 as the input port, P2 as the output port, and P3 as the isolated port. The ratio *K* is fixed as *K* = $R_{1}/R_{2}\;=$ 0.5; i.e., the radius $R_{1}$ of the ferrite material is half of the radius $R_{2}$ of the composite rod. The operating frequency was chosen as *f* = 0.4483 (*ωa*/2πc). From Figure 3 we can see that the radius $R_{2}$ of the composite rod is crucial for the performances of the PIC. The best result appears at $R_{2}=0.363a$ for the overall performance of both polarizations, with the highest output-port transmission (T2) and the lowest isolated-port transmission (T3). The TE wave is more sensitive with the change of $R_{2}$. In the following calculation, we the radius of the composite rod is fixed as $R_{2}=0.363a$.

**Figure 4:**
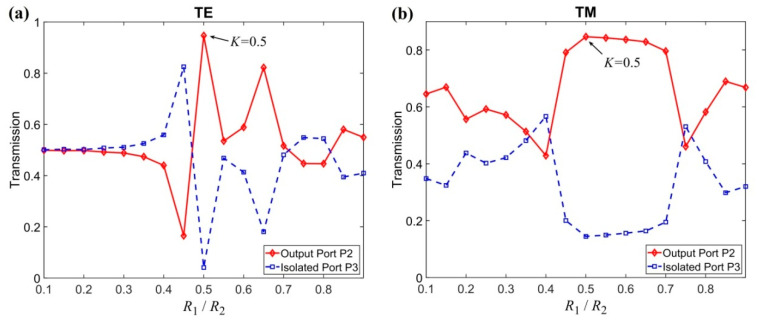
Transmissions of the output port P2 and isolated port P3 versus the radius ratio *K* = $R_{1}/R_{2}$ for (**a**) TE and (**b**) TM polarization.

**Figure 5:**
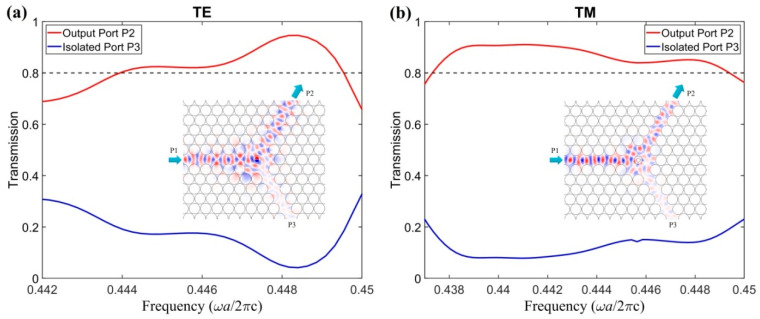
Frequency scans on transmissions of output port P2 and isolated port P3 for (**a**) TE and (**b**) TM polarization. The insets in (**a**) and (**b**) present the field distributions with input from port P1 for TE and TM waves, respectively.

The influence of the ratio *K* = $R_{1}/R_{2}$ on the performance of the PIC was then further studied, as shown in Figure 4, where the operating frequency is *f* = 0.4483 (*ωa*/2πc) and the radius of the composite rod is $R_{2}=0.363a$. It can be seen from Figure 4 that the ratio *K* is also important for the performance of the PIC. The values of T2 and T3 vary obviously with the change of ratio *K*. The best ratio is found to be *K* = 0.5 for both polarizations. The TE wave is also more sensitive with the change of *K*. In the following calculation, we fix the ratio as *K* = 0.5. The parameters of $R_{2}$ and *K* were carefully selected by considering the balance of the performances of TE and TM polarizations in which the NOM optimization method is used for obtaining these parameters. For *K* = 0.5, this means that the outside ring has a larger area than that of the inner rod. This can be understood as follows: In the field distributions shown in Figure 5, the field intensity of TE polarization in the center of the MO cavity is much higher than that away from the center, and the field distribution of TM polarization is basically uniform in the MO cavity region. Therefore, a larger area is required for the outer ring (TM-wave effective region) than that for the inner rod (TE-wave effective region) to obtain about the same circulation effect for TE and TM modes.

To further study the frequency response of the PIC, a frequency scan was performed for T2 and T3 inside the absolute PBG, as shown in Figure 5, where the radius of the composite rod is $R_{2}=0.363a$ and the ratio *K* is 0.5. It can be found from Figure 5 that, for TE polarization, the best frequency response is *f* = 0.4486 (*ωa*/2πc), with T2 = 93.9% and T3 = 4.73% (for simplicity, all output or isolated powers are represented relative to the input power). The frequency ranges from 0.4439 to 0.4496 (*ωa*/2πc) for T2 > 80%; for TM polarization, the best point is *f* = 0.4413 (*ωa*/2πc), with T2 = 91% and T3 = 7.89%. The frequency ranges from 0.4373 to 0.4494 (*ωa*/2πc) for T2 > 80%. The common frequency range for both TE and TM polarizations with T2 > 80% is from 0.4439 to 0.4496 (*ωa*/2πc). The output-port transmission of the TE wave is slightly better than that of the TM wave, but the operation bandwidth of the TE wave is a little narrower than that of the TM wave. How the transmission of the TE wave is better than the TM wave can be understood as follows: The outer ring of the composite MO rod has a larger size, and thus the outer ring will reflect/scatter more power than the inner rod. Note that the inner and outer parts of the composite MO rod are made of ferrite and plasma materials, which act mainly with TE and TM modes, respectively, so that the reflection for the TM wave is stronger than that for the TE wave in the whole MO region. As a result, the transmission of the TE wave will be better than that of the TM wave. On the other hand, one can hope to use the plasma material to construct the central part of the composite MO rod to achieve better transmission of the TM wave.

The field distributions for an input wave from P1 for TE and TM waves are displayed by the insets in Figure 5a,b, respectively (to save space, the field distributions for inputs from P2 and P3 are omitted in this case; however, complete field distributions with inputs from all ports are shown in our optimized case below). The field distributions indicate that the wave launches from the input port P1 and most of the power transmits to the output port P2. Only a small amount of power (~8%) flows to the isolated port P3. The circulator fulfills the circulation function for both polarizations. However, the output transmissions or insertion losses, especially for TM polarization, are not good enough to meet the demands for practical applications. Therefore, further optimization is necessary to obtain low insertion loss.

**Figure 6:**
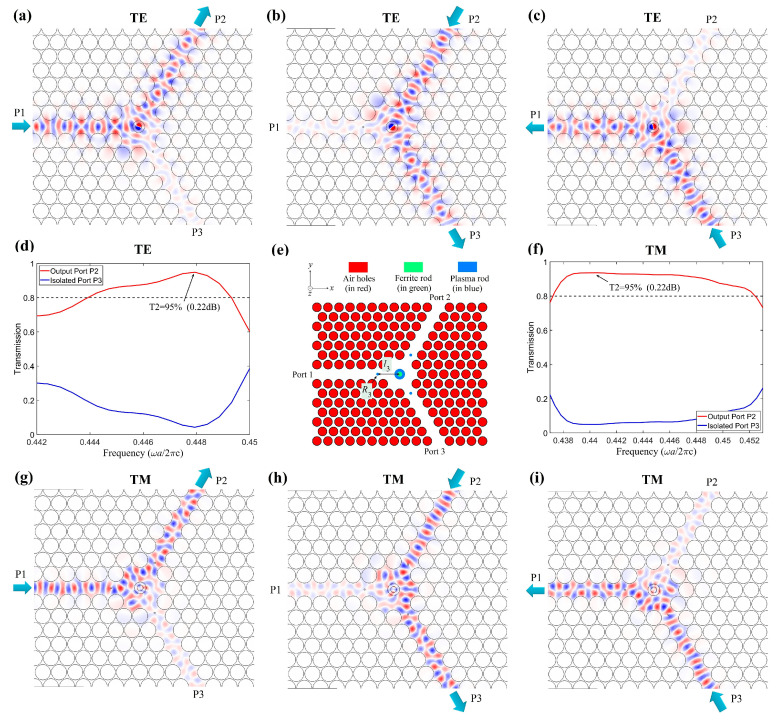
Performances and schematic of the optimized PIC. (**a**–**c**) The field distributions for TE polarization: (**a**) input from port P1, (**b**) input from port P2, and (**c**) input from port P3. (**d**,**f**) The frequency scans for (**d**) TE and (**f**) TM polarization. (**e**) The schematic of the optimized PIC. (**g**–**i**) The field distributions for TM polarization: (**g**) input from port P1, (**h**) input from port P2, and (**i**) input from port P3.

Since the performance of the PIC may be affected by the coupling condition between the central MO cavity and the waveguides, three additional small rods (formed by plasma material) were inserted into the waveguides. These additional small rods were introduced here for two reasons. The first one is that they can provide an additional rotation effect for TM polarization; the second one is that they can improve the coupling between the central MO cavity and the waveguides. The optimized model is shown in Figure 6e, where $R_{3}$ and $l_{3}$ denote the radius of the additional rods and the distance from the additional rods to the center point. It should be pointed out that we can also use rods with other shapes instead of the circular cylinders to attain our goals; however, circular cylinders are the simplest and much easier to fabricate.

It is obvious that the radius $R_{3}$ of the additional rods and the distance $l_{3}$ from the additional rods to the center point may simultaneously influence the output property of the PIC. Therefore, we should balance the performances of TE and TM polarizations and select proper $R_{3}$ and $l_{3}$ to maintain high output transmissions or insertion loss for both of them. Here, we use the NOM optimization method to optimize the parameters, which are found to be $R_{3}$ = 0.045*a* and $l_{3}$ = 1.78*a*. The performances of the optimized PIC are displayed in Figure 6, where the other parameters are the same as those of Figure 5. From the frequency scans as shown in Figure 6d,f, we can see that, for TE polarization, the best frequency response occurs at *f* = 0.4479 (*ωa*/2πc), with T2 = 95% (the corresponding insertion loss can be calculated according to 10×log(1/T2)=0.22 dB) and T3 = 3.98% (the corresponding isolation is 10×log(1/T3)=14 dB). The frequency ranges from 0.4439 to 0.4493 (*ωa*/2πc) for T2 > 80%; for TM polarization, the best output is at *f* = 0.4406 (*ωa*/2πc), with T2 = 95% (the corresponding insertion loss is 0.22 dB) and T3 = 2.4% (the corresponding isolation is 16.2 dB). The frequency ranges from 0.4372 to 0.4524 (*ωa*/2πc) for T2 > 80%. The common frequency range for both TE and TM polarizations with T2 > 80% is from 0.4439 to 0.4493 (*ωa*/2πc). The performance of the PIC, especially for the TM polarization, has been improved.

To verify the feasibility of the PIC, the field distributions are plotted in Figure 6a–c,g–i for TE and TM polarizations. As seen in these figures, for both polarizations, the input wave launches from the input port, almost all the power transmits to the output port, and only a small amount of power (~4%) flows to the isolated port. The circulator fulfills the desired circulation function for both polarizations, i.e., the circulator is a PIC.

It should be pointed out that the position of the ferrite and plasma materials in the central MO rod can also be exchanged to construct a PIC in a similar way. We also note that, although the circulator studied in this paper is designed for microwave frequencies using experimentally feasible ferrite and plasma materials due to the limit of materials at the present stage, the method for designing such a PIC can be applied to other wavebands such as an optical band when the materials required become available in the future.

## 4. Conclusions

In summary, we have proposed and demonstrated a type of PIC based on a composite rod containing ferrite and plasma materials in a 2D PhC slab. The PIC can realize simultaneously the circulation function for TE and TM polarizations while only one composite rod is necessary. In addition, three additional rods are introduced into the waveguides in our optimized model to improve the coupling condition between the center MO cavity and the waveguides. The best advantage of the proposed PIC is that it is simple and compact in structure, making it easier to integrate with other devices. The idea presented here may be useful for designing compact polarization devices in large-scale integrated photonic circuits.

## Data Availability

The data presented in this study are available on request from the corresponding author.
